# Supplementary material for: Occupational biopsychosocial factors associated with neck pain intensity, neck-disability, and sick leave: A cross-sectional study of construction labourers in an African population
Source: PLoS One. 2024 Apr 3;19(4):e0295352. doi: 10.1371/journal.pone.0295352 (PMC10990208; doi:10.1371/journal.pone.0295352)
Supplement: S1 Table — (DOCX) [file pone.0295352.s002.docx]

| **S1 Table: Collinearity diagnostics for the regression models** | | | | | | |
| --- | --- | --- | --- | --- | --- | --- |
| Model summary | | | | **Neck pain intensity** | | |
| R | **R square** | **Adjusted R square** | **Std. error of the Est.** | **variables** | **Tolerance** | **VIF** |
| .752 | .565 | .537 | 1.60806 | Weight of load (kilogram) | .921 | 1.086 |
|  |  |  |  | Load carriage frequency (number of days per week) | .143 | 6.996 |
|  |  |  |  | Duration of load carriage (number of hours per day) | .861 | 1.162 |
|  |  |  |  | Neck forward flexion | .885 | 1.130 |
|  |  |  |  | Neck backward posture | .892 | 1.121 |
|  |  |  |  | Neck twisted (rotated) posture | .962 | 1.040 |
|  |  |  |  | Neck posture frequency (number of days per week) | .148 | 6.778 |
|  |  |  |  | Neck posture duration (number of hours per day) | .943 | 1.060 |
|  |  |  |  | Break from work | .950 | 1.052 |
|  |  |  |  | Unexpected events | .878 | 1.139 |
|  |  |  |  | Order of task control | .889 | 1.125 |
|  |  |  |  | Task dependency on others | .830 | 1.205 |
|  |  |  |  | Time pressure | .801 | 1.248 |
| Model summary | | | | **Neck disability** | | |
| R | **R square** | **Adjusted R square** | **Std. error of the Est.** | **variables** | **Tolerance** | **VIF** |
| .504 | .254 | .206 | .70975 | Weight of load | .921 | 1.086 |
|  |  |  |  | Load carriage frequency | .143 | 6.996 |
|  |  |  |  | Load carriage duration | .861 | 1.162 |
|  |  |  |  | Neck forward flexion | .885 | 1.130 |
|  |  |  |  | Neck backward posture | .892 | 1.121 |
|  |  |  |  | Neck twisted (rotated) posture | .962 | 1.040 |
|  |  |  |  | Neck posture frequency | .148 | 6.778 |
|  |  |  |  | Neck posture duration | .943 | 1.060 |
|  |  |  |  | Break from work | .950 | 1.052 |
|  |  |  |  | Unexpected events | .878 | 1.139 |
|  |  |  |  | Order of task control | .889 | 1.125 |
|  |  |  |  | Task dependency on others | .830 | 1.205 |
|  |  |  |  | Time pressure | .801 | 1.248 |
| Model summary | | | | **Sick leave** | | |
| R | **R square** | **Adjusted R square** | **Std. error of the Est.** | **variables** | **Tolerance** | **VIF** |
| .250 | .063 | .003 | 2.26169 | Weight of load | .921 | 1.086 |
|  |  |  |  | Load carriage frequency | .143 | 6.996 |
|  |  |  |  | Load carriage duration | .861 | 1.162 |
|  |  |  |  | Neck forward flexion | .885 | 1.130 |
|  |  |  |  | Neck backward posture | .892 | 1.121 |
|  |  |  |  | Neck twisted (rotated) posture | .962 | 1.040 |
|  |  |  |  | Neck posture frequency | .148 | 6.778 |
|  |  |  |  | Neck posture duration | .943 | 1.060 |
|  |  |  |  | Taking additional breaks | .950 | 1.052 |
|  |  |  |  | Unexpected events | .878 | 1.139 |
|  |  |  |  | Order and pace control | .889 | 1.125 |
|  |  |  |  | Task dependency | .830 | 1.205 |
|  |  |  |  | Time pressure | .801 | 1.248 |

Taking additional breaks= Can you usually take breaks in your job in addition to the scheduled breaks? Unexpected events= Do you often find that you cannot work because of unexpected events, such as machine break down or material not delivered? Order and pace control= Can you usually control the order and pace of your tasks? Task dependency= Is the order and pace of your tasks usually dependent on others (machines, computers, customers)? Time pressure= Do you usually work under time pressure and deadlines?
