# Supplementary material for: Occupational biopsychosocial factors associated with neck pain intensity, neck-disability, and sick leave: A cross-sectional study of construction labourers in an African population
Source: PLoS One. 2024 Apr 3;19(4):e0295352. doi: 10.1371/journal.pone.0295352 (PMC10990208; doi:10.1371/journal.pone.0295352)
Supplement: Attachment — Submitted filename: Response to Reviewers .docx [file pone.0295352.s003.docx]

PONE-D-23-38173

Occupational biopsychosocial factors associated with neck pain intensity, neck-disability, and sick leave: a cross-sectional study of construction labourers in an African population

## PLOS ONE **Editor’s comments**

Dear Dr. Igwesi-Chidobe,

Thank you for submitting your manuscript to PLOS ONE. After careful consideration, we feel that it has merit but does not fully meet PLOS ONE’s publication criteria as it currently stands. Therefore, we invite you to submit a revised version of the manuscript that addresses the points raised during the review process.

Please submit your revised manuscript by Feb 11 2024 11:59PM. If you will need more time than this to complete your revisions, please reply to this message or contact the journal office at [plosone@plos.org](mailto:plosone@plos.org). When you're ready to submit your revision, log on to [https://www.editorialmanager.com/pone/](https://www.editorialmanager.com/pone/) and select the 'Submissions Needing Revision' folder to locate your manuscript file.

Please include the following items when submitting your revised manuscript:

- A rebuttal letter that responds to each point raised by the academic editor and reviewer(s). You should upload this letter as a separate file labeled 'Response to Reviewers'.
- A marked-up copy of your manuscript that highlights changes made to the original version. You should upload this as a separate file labeled 'Revised Manuscript with Track Changes'.
- An unmarked version of your revised paper without tracked changes. You should upload this as a separate file labeled 'Manuscript'.

If you would like to make changes to your financial disclosure, please include your updated statement in your cover letter. Guidelines for resubmitting your figure files are available below the reviewer comments at the end of this letter.

If applicable, we recommend that you deposit your laboratory protocols in [protocols.io](http://protocols.io) to enhance the reproducibility of your results. Protocols.io assigns your protocol its own identifier (DOI) so that it can be cited independently in the future. For instructions see: [https://journals.plos.org/plosone/s/submission-guidelines#loc-laboratory-protocols](https://journals.plos.org/plosone/s/submission-guidelines#loc-laboratory-protocols). Additionally, PLOS ONE offers an option for publishing peer-reviewed Lab Protocol articles, which describe protocols hosted on [protocols.io](http://protocols.io). Read more information on sharing protocols at [https://plos.org/protocols?utm_medium=editorial-email&utm_source=authorletters&utm_campaign=protocols](https://plos.org/protocols?utm_medium=editorial-email&utm_source=authorletters&utm_campaign=protocols).

We look forward to receiving your revised manuscript.

Kind regards,

Mohammad Mofatteh, PhD, MPH, MSc, PGCert TLHE, BSc (Hons)
Academic Editor
PLOS ONE

Journal Requirements:

When submitting your revision, we need you to address these additional requirements.

1. Please ensure that your manuscript meets PLOS ONE's style requirements, including those for file naming. The PLOS ONE style templates can be found at 
[https://journals.plos.org/plosone/s/file?id=wjVg/PLOSOne_formatting_sample_main_body.pdf](https://journals.plos.org/plosone/s/file?id=wjVg/PLOSOne_formatting_sample_main_body.pdf) and 
[https://journals.plos.org/plosone/s/file?id=ba62/PLOSOne_formatting_sample_title_authors_affiliations.pdf](https://journals.plos.org/plosone/s/file?id=ba62/PLOSOne_formatting_sample_title_authors_affiliations.pdf)

2. Thank you for stating the following financial disclosure: 
"The lead author was partly supported with personal grant from the 2020 Nature Research Award for Driving Global Impact."
Please state what role the funders took in the study.  If the funders had no role, please state: ""The funders had no role in study design, data collection and analysis, decision to publish, or preparation of the manuscript."" 
If this statement is not correct you must amend it as needed. 
Please include this amended Role of Funder statement in your cover letter; we will change the online submission form on your behalf.

3. We note that you have indicated that there are restrictions to data sharing for this study. For studies involving human research participant data or other sensitive data, we encourage authors to share de-identified or anonymized data. However, when data cannot be publicly shared for ethical reasons, we allow authors to make their data sets available upon request. For information on unacceptable data access restrictions, please see [http://journals.plos.org/plosone/s/data-availability#loc-unacceptable-data-access-restrictions](http://journals.plos.org/plosone/s/data-availability#loc-unacceptable-data-access-restrictions).

Before we proceed with your manuscript, please address the following prompts:

a) If there are ethical or legal restrictions on sharing a de-identified data set, please explain them in detail (e.g., data contain potentially identifying or sensitive patient information, data are owned by a third-party organization, etc.) and who has imposed them (e.g., a Research Ethics Committee or Institutional Review Board, etc.). Please also provide contact information for a data access committee, ethics committee, or other institutional body to which data requests may be sent.

b) If there are no restrictions, please upload the minimal anonymized data set necessary to replicate your study findings to a stable, public repository and provide us with the relevant URLs, DOIs, or accession numbers. Please see [http://www.bmj.com/content/340/bmj.c181.long](http://www.bmj.com/content/340/bmj.c181.long)for guidelines on how to de-identify and prepare clinical data for publication. For a list of recommended repositories, please see [https://journals.plos.org/plosone/s/recommended-repositories](https://journals.plos.org/plosone/s/recommended-repositories). You also have the option of uploading the data as Supporting Information files, but we would recommend depositing data directly to a data repository if possible.

Please update your Data Availability statement in the submission form accordingly.

4. Please include captions for your Supporting Information files at the end of your manuscript, and update any in-text citations to match accordingly. Please see our Supporting Information guidelines for more information: [http://journals.plos.org/plosone/s/supporting-information](http://journals.plos.org/plosone/s/supporting-information).

5. Please review your reference list to ensure that it is complete and correct. If you have cited papers that have been retracted, please include the rationale for doing so in the manuscript text, or remove these references and replace them with relevant current references. Any changes to the reference list should be mentioned in the rebuttal letter that accompanies your revised manuscript. If you need to cite a retracted article, indicate the article’s retracted status in the References list and also include a citation and full reference for the retraction notice.

## **Response to Editor’s comments**

1. I have now reformatted the manuscript to meet PLOS ONE's style requirements. Changes are in red text within the manuscript.

2. I have now amended the role of funder statement and included this in the cover letter as well as in the manuscript in line with your suggestions. Changes are in red text.

3. a. I have now clarified that due to ethical reasons, data sets can be made available upon request to the Health Research Ethics Committee of the University of Nigeria Teaching Hospital (unth.edu.ng) or to the corresponding author. Changes are in red text. 3b. does not apply. I have now updated my Data Availability statement in the submission form accordingly.

4. I have now included captions for my supporting information files at the end of the manuscript and have now updated the in-text citations to match accordingly following the guidelines you provided.

5. I have now reviewed my reference list and I can confirm that it is now complete. I can confirm that I did not cite papers that have been retracted rather I have cited our recently completed study which is at the final stages of peer review in The Journal of Pain. I have now updated the reference list to reflect this.

## **Reviewers’ comments**

[Note: HTML markup is below. Please do not edit.]

Reviewers' comments:

Reviewer's Responses to Questions

**Comments to the Author**

1. Is the manuscript technically sound, and do the data support the conclusions?

The manuscript must describe a technically sound piece of scientific research with data that supports the conclusions. Experiments must have been conducted rigorously, with appropriate controls, replication, and sample sizes. The conclusions must be drawn appropriately based on the data presented.

Reviewer #1: Yes

Reviewer #2: Yes

Reviewer #3: Yes

2. Has the statistical analysis been performed appropriately and rigorously?

Reviewer #1: Yes

Reviewer #2: Yes

Reviewer #3: Yes

3. Have the authors made all data underlying the findings in their manuscript fully available?

The [PLOS Data policy](http://www.plosone.org/static/policies.action#sharing) requires authors to make all data underlying the findings described in their manuscript fully available without restriction, with rare exception (please refer to the Data Availability Statement in the manuscript PDF file). The data should be provided as part of the manuscript or its supporting information, or deposited to a public repository. For example, in addition to summary statistics, the data points behind means, medians and variance measures should be available. If there are restrictions on publicly sharing data—e.g. participant privacy or use of data from a third party—those must be specified.

Reviewer #1: Yes

Reviewer #2: Yes

Reviewer #3: Yes

4. Is the manuscript presented in an intelligible fashion and written in standard English?

PLOS ONE does not copyedit accepted manuscripts, so the language in submitted articles must be clear, correct, and unambiguous. Any typographical or grammatical errors should be corrected at revision, so please note any specific errors here.

Reviewer #1: Yes

Reviewer #2: Yes

Reviewer #3: Yes

5. Review Comments to the Author

Please use the space provided to explain your answers to the questions above. You may also include additional comments for the author, including concerns about dual publication, research ethics, or publication ethics. (Please upload your review as an attachment if it exceeds 20,000 characters)

Reviewer #1: The article is interesting and relevant. The authors took great pains to include people who often do not participate easily in scientific research. As a result, interesting insights have emerged. Further, as far as I can judge, the statistics are adequate and the resulting insights are discussed clearly and in detail.

Reviewer #2: Thank you for giving me the opportunity to review this manuscript. The authors investigated the occupational biomechanical and occupational psychosocial factors associated with neck pain intensity, neck disability and sick leave amongst construction labourers in an urban Nigerian population. This study was well designed and the paper was written well.

Reviewer #3: This is an interesting and novel study that is relevant to readers in several fields including occupational health, pain management, musculoskeletal medicine, etc. The writing style is generally clear, and the methodology seems sound.
The authors do acknowledge some limitations. They studied men in the construction industry. In a future study, it would be very important to study women in Nigeria and other African countries who routinely carry heavy loads on their heads to and from markets and for daily tasks such as carrying water or laundry.
The comparisons to high income countries and countries with more expansive health and disability systems are noteworthy.
The article is pretty dense with detail and has a large number of tables. While the analyses in the tables are relevant and likely of interest to readers in their specific subfield, the authors might consider replacing one or two tables with figures that would help engage the more general readers.

## **Response to Reviewers’ comments**

Thank you to Reviewer 1, Reviewer 2, and Reviewer 3 for the positive comments.

In response to reviewer 3’s further comments,

I have now included that ‘future studies need to investigate women in Nigeria and other African countries who routinely carry heavy loads on their heads to and from markets and farms, and for daily tasks including carrying water from streams for domestic use such as cooking and laundry’ in the implications for future research subsection of the discussion section.

I have now moved the previous Table 6 to become a supplementary Table renamed S1 Table. The manuscript currently has 2 figures. The other tables are best illustrated in Tabular form to be more informative for future primary and secondary research. All changes are in red text within the manuscript.

## **Response to other comments**

6. I am happy for the peer review history of this manuscript with PLOS ONE to be published.

others: I have now aligned the Figures to the journal specifications and have used the Preflight Analysis and Conversion Engine (PACE) digital diagnostic tool, <https://pacev2.apexcovantage.com/> following the guidelines that you provided to ensure that this is the case.
